# Liaison psychiatry for older adults in the general hospital: service activity, development and outcomes

**DOI:** 10.1192/bjb.2017.9

**Published:** 2018-02

**Authors:** Fedza Mujic, Ruth Cairns, Vivienne Mak, Clare Squire, Andrew Wells, Ahmed Al-Harrasi, Martin Prince

**Affiliations:** 1 South London and Maudsley NHS Foundation Trust, UK; 2 Institute of Psychiatry, London

## Abstract

**Aims and method:**

This study used data collected to describe the activity, case-load characteristics and outcome measures for all patients seen during a 6-year period.

**Results:**

The service reviewed 2153 patients over 6 years with referral rates and case-load characteristics comparable to those described in a previous study period. The team saw 82% of patients on the day they were referred. Data and outcome measures collected showed significant complexity in the cases seen and statistically significant improvement in Health of the Nation Outcome Scales (HoNOS) scores following service input.

**Clinical implications:**

The outcome measures used were limited, but the study supports the need for specialist liaison psychiatry for older adults (LPOA) services in the general hospital. The Framework of Outcome Measures – Liaison Psychiatry has now been introduced, but it remains unclear how valid this is in LPOA. It is of note that cost-effectiveness secondary to service input and training activities are not adequately monitored.

**Declaration of interest:**

None.

Around two-thirds of National Health Service (NHS) in-patient beds are occupied by older people, with up to 60% having an existing mental disorder or developing one during their admission.^1^ Medical in-patients are three to four times more likely to develop a mental illness than the healthy population, with high prevalence rates of depression in patients with diabetes and coronary heart disease.^2^ High rates of dementia, delirium and depression have been reported in older in-patients.^3^ It is important to identify and treat these conditions, as mental illness has been shown to increase the risk of poor health outcomes, including loss of independence, reduced life expectancy and increased mortality.^3^^–^^5^

Early studies showed that around 30% of all liaison psychiatry referrals were for people over the age of 65.^6^ Despite an ageing population and strong arguments for specialist liaison old age psychiatry input in general hospitals, a report in 2003 showed that 73% of UK services were provided through a traditional sector-based model,^7^ and it is not clear to what extent this has changed. Recently, the rapid assessment and integrated discharge (RAID) liaison psychiatry service led to significant savings by reducing readmissions and length of stay.^8^ Notably, elderly people represented about a third of the sample but accounted for around 90% of savings through reduced bed use.

The aim of this study was to provide an in-depth analysis of a well-established liaison psychiatry for older adults (LPOA) service, regarding its activity, case-load characteristics and use of outcome measures.

## Method

### Setting and team development

King's College Hospital is a 950-bed London teaching hospital covering a large inner-city catchment area. The LPOA service was restructured in 2000, moving from an *ad hoc* off-site service to a dedicated specialist service with staff based in the hospital. Also, the old consultation model of service delivery was changed to a true liaison model in December 2000 as previously described,^9^ but has been focused on in-patients over the age of 65 years. No older adult specialist cover had been provided to the emergency department until early 2016, owing to the small team size and insufficient resources.

The staffing level of the service had been unchanged for around 10 years from 2000 to 2010, with limited (approximately two sessions) honorary consultant psychiatrist input, a full-time staff grade/associate specialist doctor, a full-time junior doctor, and no or very poor administrative support. Attempts to improve the staff skill mix of this strongly doctor-led service failed for a long time, owing to resource issues and cuts within the mental health trust. However, with the help of the acute trust providing funding for 18 months initially, the service employed a clinical nurse specialist (CNS) in late 2010. This post has continued and is now funded by the mental health trust. Funding was also secured for a half-time administrator in 2015 and, as the team has expanded, the CNS role has been extended and now also operates as the team manager.

Between October 2013 and September 2015, consultant cover for the service was provided by the substantive consultant from another older adult liaison service within the trust. Funding for a half-time substantive consultant psychiatrist was finally agreed and commenced in September 2015. Since then, and owing to increased demands on the service, there have been further developments to the team skill mix, including fixed-term funding (now made recurrent) for a band 6 psychiatric liaison nurse (PLN) to cover in-patients and Core-24 NHS England (NHSE) funding (https://www.england.nhs.uk/mental-health/adults/crisis-and-acute-care/transformation-funding/) for a CNS to provide specialist old age psychiatry input to the emergency department, and a part-time trainee psychologist has joined the team. The most recent developments are further NHSE Core-24 funding for a clinical psychologist and occupational therapist within the liaison psychiatry department; both posts will work across the general adult and older adult teams. The team has now become much more integrated with the general adult psychiatry team, with a single point of access and closer collaboration.

### Service activity

The activity of the service has been monitored extensively since its inception in December 2000. This has included descriptive data about the source of and reason for referrals, characteristics of the case-load, outcome of the team's assessment, outcomes and follow-up arrangements. The rationale for this has been to gather information to support further service development and to identify any problems that needed addressing. The data collection has been anonymised throughout, with no patient identifiers recorded on the electronic database used for further analysis.

### Sample and data collection

The study included all patients seen by the King's College Hospital LPOA service between January 2010 and December 2015.

A two-page form was filled in by the assessing clinician for each patient, containing demographics, reason for referral, response time, outcome of the psychiatric assessment, discharge destination and follow-up arrangements. Data about mental capacity assessment were also collected, and Mini-Mental State Examination (MMSE) scores^10^ were recorded where possible. The main performance and outcome data collected included response time (and whether target times were met) and Health of the Nation Outcome Scales for Elderly People (HoNOS 65+) © Royal College of Psychiatrists 1999.^11^ The response time standards set for the service were assessment of all urgent referrals within 24 h, medium-urgency referrals within 3 days, and low-urgency referrals within 5 days. A second HoNOS 65+ rating was completed for patients under the care of the service for 2 weeks or longer. A record was also kept of new diagnoses of dementia and antipsychotic medication reviews in patients with dementia. The information from each form was anonymised and transferred on to the electronic database by F.M.

### Statistical analysis

Data were analysed using SPSS version 21.0. Descriptive statistics were used to analyse the main demographic characteristics of the case-load (age, gender, ethnicity), clinical characteristics, cognitive status (MMSE score) and outcome data of the patients in the sample. We also used paired *t*-tests to test the difference between paired HoNOS 65+ ratings.

## Results

### Case-load

The service reviewed 2153 patients during the studied period, amounting to approximately 360 patients seen on an annual basis. This did not include referred patients where the liaison service provided advice and information only, or where the referral was deemed more appropriate for another team.

The majority of patients were admitted from their homes (*n* = 1940; 90.1%), while only a minority were either admitted from care homes or transferred from another hospital. Apart from medical and surgical issues, 435 (20.2%) patients in this sample were admitted to hospital owing to falls, with 124 (28.5%) of these sustaining various fractures. Suicide attempts, overdose or other self-harm incidents were the reason for admission for 68 (3.1%) patients. The main characteristics of patients and referrals are outlined in Table 1.

**Table 1:** Main characteristics of the referrals

Age, mean years (s.d.)	78.6 (7.93); min 60, max 106
Gender	
Female	1141 (53%)
Male	1011 (47%)
Ethnicity	
White European	1603 (74.5%)
Caribbean	313 (14.5%)
African	99 (4.6%)
Asian	68 (3.2%)
White other	46 (2.1)
Other	24 (1.1)
Referred by	
Geriatrics	598 (27.5%)
Acute medicine	564 (26.2%)
Acute medical unit	378 (17.6%)
Orthopaedics	94 (4.4%)
Other surgeons	152 (7.1%)
Other	367 (17.0%)
Referral urgency	
High	758 (35.2%)
Medium	1292 (60.0%)
Low	103 (4.8%)

### Referral requests and assessment

The vast majority of referrals were for advice on issues affecting the patient's stay in the hospital, including mental health diagnosis (82.5%) and management (90.5%). Advice on mental capacity assessment was requested in 8.5% of the referrals, which is lower than when the service was first established and before the Mental Capacity Act 2005 was introduced.^12^ Other requests were mainly related to patients' discharge arrangements and made a smaller contribution to the total number of referrals, e.g. advice on placement (1%) and mental health follow-up (5.1%).

The main presenting problems that triggered referral were low mood (65.8%), impaired cognition and confusion (36.2%), behavioural disturbance (21.7%), and abnormal beliefs and experiences (15.4%). The most specific referral questions asked were related to mental state (92.5%) and medication (52%), while other questions included issues with cognition (8.2%) and suicidality (11%).

The majority of patients were diagnosed with one or more psychiatric disorders. Only a small number of patients did not have any psychiatric diagnosis following the assessment (4.3%). The diagnoses are outlined in Table 2.

**Table 2:** Case-load

Diagnosis	Main (*n* = 2153)	Second (*n* = 559)	Overall frequency of diagnosis (*n* = 2016)
Delirium	473 (22.0%)	187 (33.5%)	660 (32.7%)
Dementia	438 (20.3%)	113 (20.2%)	551 (27.3%)
Adjustment disorder	483 (22.4%)	46 (8.2%)	529 (26.2%)
Depression	307 (14.3%)	57 (10.2%)	364 (18.1%)
Psychotic illness	125 (5.8%)	29 (5.2%)	154 (7.6%)
Alcohol	35 (1.6%)	26 (4.7%)	61 (3.0%)
Bipolar affective disorder	17 (0.8%)	8 (1.4%)	25 (1.2%)
Other	183 (8.6%)	93 (16.6%)	276 (13.7%)
No diagnosis	92 (4.3%)	n/a	n/a

### Interventions and outcomes

**Fig. 1:**
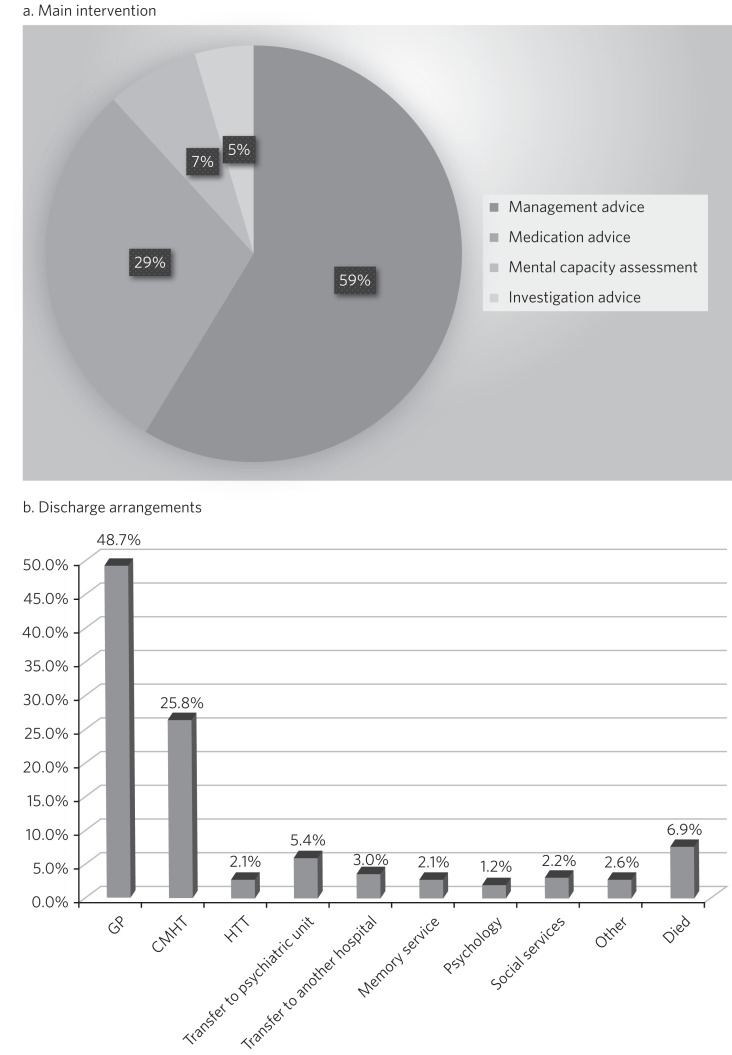
The main interventions and discharge arrangement. (a) Main intervention. (b) Discharge arrangements.

In most cases, the referring team received advice on the patient's ongoing management and medication (Fig. 1a). No further intervention was provided in 546 (25.4%) of cases, while others received some further interventions from the service. These included transfers to a mental health unit (*n* = 98, 4.6%); referral for community mental health team (CMHT) follow-up (*n* = 249, 11.6%); referral to the Mental Health for Older Adults home treatment team (HTT; *n* = 45, 2.1%); and referral for psychological intervention or assessment (*n* = 21, 1.0%). Mental capacity was assessed in a total of 12.2% of patients (as the main intervention in 7% and as an additional intervention in the remaining 5.2% of cases).

Following discharge from hospital, most patients returned to their homes (62.3%) and were followed up by their general practitioner (GP; 48.7%) (Fig. 1b). Of the total number of patients, 112 (5.2%) were discharged into an EMI (elderly mentally infirm) care home.

### Service activity and outcome measures

As shown in Table 3, the service was highly responsive, particularly for urgent referrals, for which almost all patients were seen on the same day and many within 4 h. The achievement was lower for medium-urgency referrals (95.2% seen within 3 days) and low-urgency referrals (93.2% seen within 5 days).

**Table 3:** Service responsiveness, contacts and HoNOS rating

Referrals (*n* = 2153)	Seen within 1 day	Seen within 4 h
High	754 (99.5%)	754 (99.5%)
Medium	969 (75.1%)	493 (38.3%)
Low	36 (35.0%)	19 (18.4%)
All referrals	1759 (81.7%)	1097 (50.9%)
Contacts		
All patients (*n* = 2153)		
Single assessment	1141 (53.0%)	
Mean time under care (days)	9.7 (s.d. 15.23)	
Average number of contacts	2.6 (s.d. 3.269)	
Mean time spent with patient	152.3 min. (s.d. 170.894)	
Follow-up patients (*n* = 1012)		
Mean time under care (days)	17.06 (s.d. 19.550)	
Mean number of contacts	4.4 (s.d. 4.070)	
Mean time spent with patient	228.84 min (s.d. 221.971)	
HoNOS 65+ rating		
Mean initial HoNOS 65+ rating (*N* = 1081)	12.53 (s.d. 4.125); min 1, max 32	
Mean paired HoNOS 65+ ratings (*N* = 230)		
Initial	14.65 (s.d. 3.919)	
On discharge	11.80 (s.d. 4.599)	
Difference – paired *t*-test:	10.035 (d.f. 229); *P* < 0.001	
Difference – 95% CI	2.296–3.418	

Table 3 shows that for those patients who were seen on more than one occasion, the service had a substantial number of contacts, and significant time was spent on their mental healthcare during their stays in the hospital.

The HoNOS 65+ rating scale was introduced in January 2013 as one of the outcome measures for the service. Analysis of this data (Table 3) indicates a significant degree of complexity of the cases seen by the service and a statistically significant improvement in HoNOS scores following input from the service.

### Good clinical practice

New diagnoses of dementia made by the service were included in data collection from January 2013, and a record of antipsychotic reviews in people with dementia started in May 2013. Between January 2013 and December 2015, 273 patients with no existing dementia diagnosis (21.6% of all referrals) were identified with probable dementia that needed further assessment, and 51 patients (4.0% of all referrals) were diagnosed with a new diagnosis of dementia by the liaison service. Of 50 patients with dementia who were on antipsychotic medication at the time of referral, only one patient (2%) did not have his medication reviewed by the service.

## Discussion

The results of this study indicate that the LPOA service at King's College Hospital, London, has continued to play an important part in mental health provision to adults aged 65 and over admitted to general hospital beds since its initial description.^9^ The total numbers of patients seen per year, their demographic characteristics and case mix have all remained comparable to those described in 2000/2001. The service has also maintained a good responsiveness, particularly for urgent referrals which, although not described in this data set, the service has extended into the emergency department and clinical decisions unit during this study period.

There are two significant points that have changed in service activity since the last review. First, there has been a marked increase in the number of patients with delirium seen by the service; second, there has been a significant decrease in the number of referrals for assessment of mental capacity. The first point is likely to be explained by new developments within the general hospital and the close relationship of the liaison service with the King's delirium and dementia team that was established in January 2013. This may have led to better recognition of delirium and targeted involvement of liaison psychiatry in management of complex cases for which there is diagnostic uncertainty requiring expertise in recognition, or where pharmacological management is required owing to risks to patients and others. The second point is likely to reflect increased awareness and improved skills of medical and surgical teams in assessing mental capacity following the introduction of the Mental Capacity Act 2005 and the increased training that followed this legislation. Our service has retained an important role in providing a second opinion in complex capacity assessments.

The results presented suggest that the service has played a part in discharge facilitation, with the majority of patients returning home and being followed up by their GP. Relatively small numbers of patients required referral to the CMHT, and for those already under CMHT care there was good liaison between respective mental health services. Only a small percentage of patients required transfer to psychiatric units, but this may have also been influenced by the development of an older adults' HTT in the last 3 years of the study period.

The National Dementia Strategy has included good quality of care within general hospitals as one of its key objectives for patients with dementia.^13^ Around 27.3% of patients seen had a diagnosis of dementia during the study period. This is in line with previous estimates that, at any time, up to a quarter of older patients in general acute hospitals will have dementia.^14^ Fifty-one patients were diagnosed with a new diagnosis of definitive dementia from January 2013 and included in the total number of cases. A further 20% of patients seen during the 3-year study period were suspected to suffer from dementia, with a discharge recommendation for further assessment. These data, and the fact that all but one patient with dementia and on antipsychotic medication had this reviewed by the service, suggest that the service engaged in important local and national initiatives for dementia diagnosis and care.

Until the recent introduction of the Framework for Routine Outcome Measurement in Liaison Psychiatry (FROM-LP), there had been no consensus on how to best capture the diverse activities, outcomes and performance of liaison psychiatry services.^15^ In addition, as was the case with this team, teams have often had small numbers of staff and limited administrative support, also limiting their capacity for routine outcome measure collection. Despite this, we have recognised the importance of monitoring activity in relation to further team development and during the study period have recorded referral response times and clinician-rated HoNOS 65+ outcome scores for all patients seen. Average HoNOS 65+ scores indicated that the patients seen had conditions of moderate severity and complexity, and a statistically significant improvement was seen in the patients for whom paired ratings were done. Although these are positive data, the extent to which the physical health domains and improvements contributed to positive outcomes is unclear, and this itself does not fully reflect all aspects of the service's activities and roles within the general hospital.

A recent review suggested that the FROM-LP is a very useful tool to measure service quality and clinical effectiveness, and represents a significant step towards developing nationally unified outcome measures.^16^ There is also an increasing expectation that outcome measures are available to secure funding and support liaison psychiatry service growth. In response to this, we have been collecting outcome measures as suggested by FROM-LP,^17^ as well as HoNOS 65+, since January 2016. However, from the outcome data collected so far, we have some concerns about the validity of the measures and also that they do not measure the aspects of LPOA services that have previously been shown to make them cost-effective. Economic analysis of the Birmingham RAID service suggested that elderly people in their sample accounted for around 90% of total savings with reduced bed usage. We therefore suggest that this is of particular importance for older adult liaison services, and that there is a strong economic case for targeting increased resources for this patient group. This would also support the case for developing specialist LPOA teams as a part of the national strategy for improved liaison psychiatry services within the general hospital.

### Strengths and limitations of the study

The strength of this study is that it provides a large data-set and reflects the everyday practice of a busy inner-city LPOA service. There is very little missing data, as collection was overseen and coordinated by a single practitioner who ensured that data were collected for all patients seen. As such, the study included a large number of patients, ensuring an adequate statistical power. It also gives a clear indication of the need for specific older adults' liaison teams, and indicates the scope for further development and analysis of outcome measures to support this and the cost-effectiveness of services.

This is a descriptive study and does not intend to compare the King's College liaison service for older people with other liaison service provision models for older adults. It is possible that an awareness of service monitoring by team members who were also responsible for data collection might have had an impact on the care provided, but arguably this is less likely as data collection is now a routine part of clinical practice within the team, and there is increasing emphasis on outcome measures and quality improvement activities. Another possible limitation is that the activities and outcomes of the team described in this paper may not be generalisable to other LPOA teams in the UK where the team structure or patient demographics differ.

### Clinical implications

The results of this study support the need for specialist LPOA services for older people admitted to general hospitals. However, finding easily measurable outcomes of liaison psychiatry services remains a challenge if we are to find valid measures that also support the services in terms of identifying necessary developments and growth. The King's College Hospital LPOA team is now using the FROM-LP and will pilot the use of these outcome measures. However, we suggest that there are other aspects of service activity in older adult liaison that are not adequately monitored or audited using this framework. For this reason, we continue to use HoNOS 65+, as we feel that this provides a measure of complexity and also indicates improvement over time with paired scores. In terms of further development of outcome indicators for LPOA, we feel it is important to consider whether length of time from admission to referral to liaison affects overall length of stay, as this could help to provide information about cost-effectiveness of older adult liaison services. Similarly, we feel it will be important to measure the impact of training activities for general hospital staff in terms of raised awareness and timely referrals to the service.

## About the authors

**Fedza Mujic** is an associate specialist in liaison psychiatry for older people, South London and Maudsley NHS Foundation Trust; **Ruth Cairns** is a consultant in liaison psychiatry for older people, South London and Maudsley NHS Foundation Trust; **Vivienne Mak** is a consultant in liaison psychiatry for older people, South London and Maudsley NHS Foundation Trust; **Clare Squire** is a clinical nurse specialist in liaison psychiatry for older people, South London and Maudsley NHS Foundation Trust; **Andrew Wells** is a psychiatric liaison nurse, South London and Maudsley NHS Foundation Trust; **Ahmed Al-Harrasi** is an ST5 in South London and Maudsley NHS Foundation Trust; **Martin Prince** is a professor of epidemiological psychiatry and head of the Department of Health Service and Population Research at the Institute of Psychiatry.
